# Radiosensitization of HSF-1 Knockdown Lung Cancer Cells by Low Concentrations of Hsp90 Inhibitor NVP-AUY922

**DOI:** 10.3390/cells8101166

**Published:** 2019-09-28

**Authors:** Annett Kühnel, Daniela Schilling, Stephanie E. Combs, Bernhard Haller, Melissa Schwab, Gabriele Multhoff

**Affiliations:** 1 Department of Radiation Oncology, Klinikum rechts der Isar, Technische Universität München (TUM), Ismaninger Straße 22, 81675 Munich, Germany; annett.kuehnel@tum.de (A.K.); daniela.schilling@tum.de (D.S.); stephanie.combs@tum.de (S.E.C.); 2 Institute of Radiation Medicine (IRM), Department of Radiation Sciences (DRS), Helmholtz Zentrum München, Ingolstädter Landstraße 1, 85764 Neuherberg, Germany; 3 Deutsches Konsortium für Translationale Krebsforschung (DKTK), Partner Site Munich, 81675 Munich, Germany; 4 Institute for Medical Informatics, Statistics and Epidemiology, Technische Universität München, Klinikum rechts der Isar, Ismaningerstr. 21, 81675 Munich, Germany; bernhard.haller@mri.tum.de; 5 Center for Translational Cancer Research, Technische Universität München (TranslaTUM), Klinikum rechts der Isar, Einsteinstr. 25, 81675 Munich, Germany; mellissa.schwab@tum.de

**Keywords:** heat shock factor (HSF-1) knockdown, heat shock proteins 70 and 27, radiosensitization, Hsp90 inhibitor NVP-AUY922, homologous recombination (HR)

## Abstract

The inhibition of heat shock protein 90 (Hsp90) a molecular chaperone for multiple oncogenic client proteins is considered as a promising approach to overcome radioresistance. Since most Hsp90 inhibitors activate HSF-1 that induces the transcription of cytoprotective and tumor-promoting stress proteins such as Hsp70 and Hsp27, a combined approach consisting of HSF-1 knockdown (k.d.) and Hsp90 inhibition was investigated. A specific HSF-1 k.d. was achieved in H1339 lung cancer cells using RNAi-Ready pSIRENRetroQ vectors with puromycin resistance. The Hsp90 inhibitor NVP-AUY922 was evaluated at low concentrations—ranging from 1–10 nM—in control and HSF-1 k.d. cells. Protein expression (i.e., Hsp27/Hsp70, HSF-1, pHSF-1, Akt, ß-actin) and transcriptional activity was assessed by western blot analysis and luciferase assays and radiosensitivity was measured by proliferation, apoptosis (Annexin V, active caspase 3), clonogenic cell survival, alkaline comet, γH2AX, 53BP1, and Rad51 foci assays. The k.d. of HSF-1 resulted in a significant reduction of basal and NVP-AUY922-induced Hsp70/Hsp27 expression levels. A combined approach consisting of HSF-1 k.d. and low concentrations of the Hsp90 inhibitor NVP-AUY922 reduces the Hsp90 client protein Akt and potentiates radiosensitization, which involves an impaired homologous recombination mediated by Rad51. Our findings are key for clinical applications of Hsp90 inhibitors with respect to adverse hepatotoxic effects.

## 1. Introduction

Lung cancer is one of the most commonly diagnosed cancer in Western societies and the leading cause of cancer-related deaths worldwide [1,2]. Despite novel therapeutic approaches including immune checkpoint inhibitor blockade to which only a proportion of patients showed beneficial responses, the 5-year survival rate of late stage tumor diseases remains poor with approximately 15% [3,4]. Radio- and chemoresistance of pulmonary tumor cells is a major limiting factor for a favorable clinical outcome. Therefore, there is a high unmet medical need for combined therapeutic approaches that significantly improve the radiosensitivity of lung cancer cells.

The heat shock proteins Hsp90, Hsp70, and Hsp27 and the transcription factor heat shock factor-1 (HSF-1) are frequently overexpressed in many tumor cell types [5,6,7], including lung carcinoma. Elevated HSP levels contribute to a malignant transformation and mediate resistance to chemo- and radiotherapy by interfering with apoptotic signaling pathways [8,9]. Hsp90 is an attractive therapeutic target [10,11,12,13,14] and its inhibition affects many oncogenic driver proteins, which are involved in cell cycle regulation, apoptosis, and DNA repair [15,16,17]. High concentrations of the Hsp90 inhibitor NVP-AUY922 can increase radiosensitivity of tumor cells [14,18]. Apart from these positive effects, the inhibition of Hsp90 induces the release of HSF-1 from the Hsp90 complex and thereby stimulates the transcription of the cytoprotective chaperones Hsp70 and Hsp27 [19,20,21,22,23,24,25,26,27]. Due to the anti-apoptotic activities of Hsp70 and Hsp27 that promote tumor cell survival, the combined effects of an HSF-1 and Hsp90 inhibition were assessed on the radiosensitivity of H1339 cells.

## 2. Methods

### 2.1. Reagents and Treatment

A stock solution (10mM) of NVP-AUY922 (Novartis, Basel, Schweiz) was prepared in 100% DMSO and further diluted in PBS. A vehicle control with the respective amount of DMSO was diluted in PBS was used as an internal control. If not indicated otherwise, cells were incubated with NVP-AUY922 for 24 h.

### 2.2. Cells and Cell Culture

The human lung cancer cell line H1339 was cultured in RPMI 1640 medium (Thermo Fisher Scientific, Waltham, MA, USA) supplemented with 10% v/v heat-inactivated FCS (Sigma-Aldrich, St. Louis, MO, USA), as described previously [14,28]. The authenticity of the tumor cell lines was tested by the DSMZ (German Collection of Microorganisms and Cell Cultures, Braunschweig, Germany). Cells were routinely checked and determined as negative for mycoplasma contaminations.

### 2.3. Retroviral Vectors and Infection

HSF-1 RNAi-Ready pSIRENRetroQ vectors with puromycin resistance were used for a specific k.d. of HSF-1. Target sequence for HSF-1 small interfering RNA was 5′-TATGGACTCCAACCTGGATAA-3′ [29]. Retroviruses were produced by transfection of Phoenix cells with pSIREN-RetroQ/HSF-1 shRNA (HSF-1 k.d.) or pSIREN-RetroQ (ctrl) using Ca-phosphate. Tumor cells were infected with virus containing supernatants in the presence of 10 μg/mL polybrene and selected by puromycin (2 μg/mL).

### 2.4. Western Blot Analysis and ELISA

Cells were lysed in TBST buffer as described previously [30]. The protein content was determined using the BCA™ Protein Assay Kit (Pierce). After transfer on PVDF membranes (BioRad) blots were blocked in 5% skim milk and incubated with antibodies (4 °C, overnight) directed against HSF-1 (ADI-SPA-901; Enzo Life Sciences Inc., Farmingdale, NY, USA), HSF-1 phospho S326 (pHSF-1) (ab76076; abcam), Hsp70 (ADI-SPA-810; Enzo Life Sciences; cmHsp70.1; multimmune GmbH), Hsp27 (ADI-SPA-800; Enzo Life Sciences), Akt (Cell Signaling Technology Europe, Frankfurt, Germany) and β-actin (A5316; Sigma-Aldrich, St. Louis, MO, USA). Horseradish-peroxidase (HRP)-conjugated anti-rabbit/anti-mouse antibodies (Promega, Fitchburg, WI, USA, W401B/W402B) were used as secondary antibodies. Immune complexes as detected by ECL detection system (GE Healthcare, Chicago, IL, USA) were imaged digitally (ChemiDoc Touch Imaging System, Biorad, Hercules, CA, USA). Hsp70 protein concentrations in cell lysates were quantified by ELISA (R&D systems, Minneaplis, MN, USA) following the manufacturer’s recommendations.

### 2.5. HSE Luciferase Assay

The HSF-1 transcriptional activity of treated (100 nM NVP-AUY922, 24 h) and untreated cells was determined using the HSE luciferase assay (Qiagen, Hilden, Germany) and the Dual Glo Luciferase assay system (Promega, Fitchburg, WI, USA) following the manufacturer’s recommendations [31].

### 2.6. Proliferation Assay

Cells were seeded in 96-well plates (1000 cells/well). On the following day, cells were treated with NVP-AUY922 (2, 20, 50, 75, 100 nM) and incubated under standard conditions for 24 h and 48 h. Proliferation was measured using the colorimetric alamarBlue assay, according to manufacturer’s instructions (Thermo Fisher Scientific, Waltham, MA, USA). The proliferation rate of untreated cells was defined as 100% in each experiment.

### 2.7. Cell Death and Apoptosis Assays

Cell death and apoptosis were measured by propidium iodide (PI) active Caspase-3 Apoptosis assays (BD Pharmingen) and Annexin V-FITC staining (Roche Diagnostics, Rotkreuz, Switzerland). Cells were collected 24 h after treatment with 100 nM NVP-AUY922 or 24 h after irradiation (IR) and stained according to the manufacturer’s instructions and analyzed on a FACSCalibur flow cytometer (BD, Franklin Lakes, NJ, USA).

### 2.8. Clonogenic Survival Assay and Irradiation

Radiosensitivity was determined by using a clonogenic colony formation assay as described previously [14]. Treated (NVP-AUY922, 24 h) and vehicle (DMSO) treated cells were irradiated with the indicated doses using the RS225A device (Xstrahl Limited, Camberley, UK) at a dose rate of 1 Gy/min (70 keV, 10 mA). 1 h after irradiation, the medium was changed by a drug-free control medium. On days 7–9 after cultivation at 37 °C in 5% CO_2_ colonies were fixed in ice-cold methanol and stained with 0.1% crystal violet. All colonies containing ≥50 cells were counted automatically using an automated Bioreader^®^ system (Bio-Sys GmbH, Karben, Germany). Survival curves were fitted to the linear quadratic model using Sigmaplot (Systat Software Inc, Erkrath, Germany).

### 2.9. Cell Cycle Analysis

Cells were treated with NVP-AUY922 (10 nM, 24 h) followed by irradiation with 6Gy. 24 h after IR cells were harvested, fixed in 70% ice-cold ethanol cells, incubated in propidium iodide (PI) staining solution (PBS, 0.1% Triton X-100, 0.2 mg/mL RNase A, 0.02 mg/mL PI) for 1 h at room temperature and analyzed on a FACSCalibur flow cytometer (BD). Cell cycle distribution was determined using ModFit LT (Verity Software House, Topsham, ME, USA).

### 2.10. Alkaline Comet Assay

DNA damage was assessed by using a modified alkaline comet assay, which was originally described by Singh et al. [32,33]. The scoring of the Comet assay was performed with an epifluorescence microscope equipped with a charge-coupled device camera from Leica using the image analysis software Komet 6.0 (Andor). The results shown are derived from three independent experiments, in each experiment 100 cells were scored. 

### 2.11. Flow Cytometry of γH2AX

DNA double strand breaks (DBSs) were measured by using γH2AX (phosphorylated Histone H2AX) antibody (Alexa Fluor 488 conjugate, Novus Biologicals, Centennial, CO, USA) in control (vehicle treated) and NVP-AUY922-treated (10 nM, 24 h) cells collected 30 min and 24 h after irradiation. The detailed protocol is described by Murakami et al. [34]. The mean fluorescence intensity (mfi) of γH2AX-stained cells was determined by flow cytometry on a FACSCalibur instrument.

### 2.12. Immunostaining for 53BP1 and Rad51 Foci

One day after seeding, cells were treated with NVP-AUY922 (10 nM, 24 h) followed by irradiation with the indicated doses. 4 h and 24 h after irradiation, cells were fixed in 2% paraformaldehyde/PBS. Cell lysis was achieved by submerging the slides in lysis buffer (0.15% Triton X-100/PBS) followed by incubation in blocking buffer (1% BSA, 0.15% glycine/PBS). Then, cells were stained with 53BP1 (1:300; Novus Biologicals, Centennial, CO, USA) and Rad51 (1:300; Merck Millipore, Burlington, MA, USA) antibodies overnight at 4 °C. After washing, slides were incubated overnight with an AlexaFluor 555 secondary antibody (1:500; Thermo Fisher Scientific, Waltham, MA, USA) and, after another washing in PBS, embedded in 4′,6-diamidino-2-phenylindole (DAPI)/Vectashield (Vector Laboratories, Burlingame, CA, USA) solution. The stained foci were counted in the nucleus.

### 2.13. Statistics

Statistical analysis was performed using SPSS 18.0.2 software (IBM, Armonk, NY, USA). The Student’s *t*-test was used to evaluate significant differences (* *p* ≤ 0.05, ** *p* ≤ 0.01, *** *p* ≤ 0.001). All data were obtained from at least three independent experiments.

## 3. Results

### 3.1. HSF-1 k.d. Reduces Hsp70/Hsp27 Expression and Sensitizes Tumor Cells towards Hsp90 Inhibition

**Figure 1:**
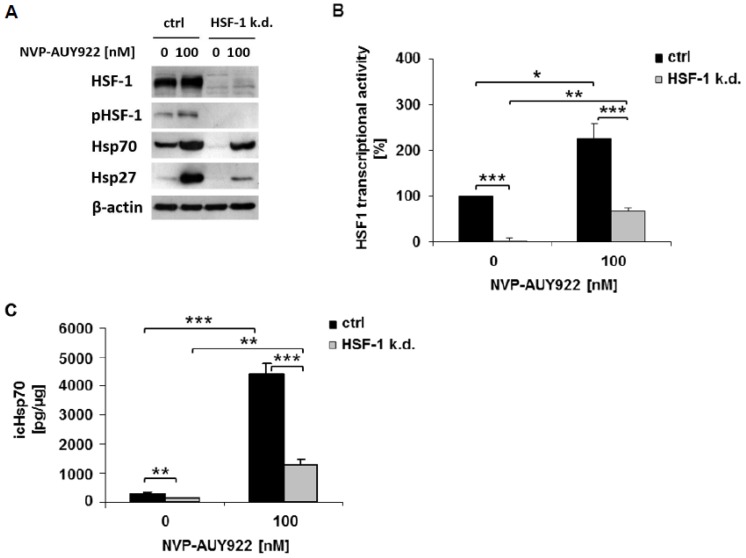
HSF-1 k.d. reduces the expression of Hsp70 and Hsp27 and the transcriptional activity of HSF-1. (**A**) Representative immunoblot showing the expression of HSF-1, HSF-1 phospho S326 (pHSF-1), Hsp70, Hsp27, and β-actin in H1339 cells transfected with control (ctrl) or HSF-1 shRNA (HSF-1 k.d.). Cells were treated with NVP-AUY922 (100 nM) for 24 h. (**B**) Transcriptional activity of an HSF-1 responsive firefly luciferase construct in H1339 ctrl and HSF-1 k.d. cells. Cells were treated with NVP-AUY922 (100 nM) for 24 h. Significance * *p* ≤ 0.05; ** *p* ≤ 0.01; *** *p* ≤ 0.001. (**C**) Intracellular (ic) Hsp70 protein concentrations assessed by ELISA in H1339 ctrl and HSF-1 k.d. cells treated with NVP-AUY922 (100 nM) for 24 h. Significance * *p* ≤ 0.05; ** *p* ≤ 0.01; *** *p* ≤ 0.001.

HSF-1 was specifically knocked down in H1339 cells by transfection with shRNA (HSF-1 k.d.). As a control, H1339 cells were transfected with an empty plasmid vector (ctrl). HSF-1 k.d. in H1339 cells was verified by a drastic reduction in the total amount of non-phosphorylated (HSF-1) and phosphorylated HSF-1 (pHSF-1) protein (Figure 1A), and a significant downregulation of the basal and NVP-AUY922-induced transcriptional activity of HSF-1, as compared to control cells (Figure 1B). The activity of NVP-AUY922 was verified by significantly upregulated intracellular Hsp70 and Hsp27 levels in control cells (Figure 1A). In HSF-1 k.d. cells the Hsp70 and Hsp27 levels increased only marginally upon NVP-AUY922 treatment (Figure 1A). Basal as well as NVP-AUY922-induced Hsp70 concentrations, as determined by ELISA, were significantly found to be reduced in HSF-1 k.d. cells compared to control cells (Figure 1C).

**Figure 2:**
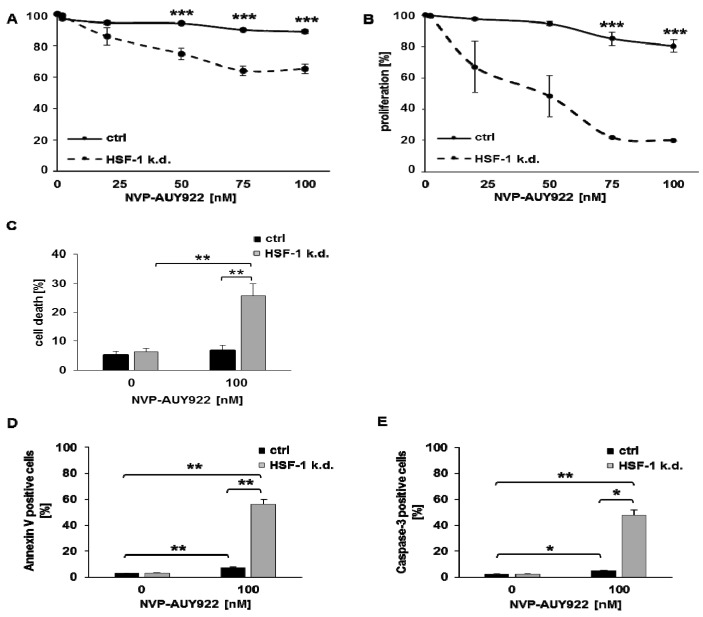
Hsp90 inhibition significantly inhibits proliferation and induces apoptosis in HSF-1 k.d. cells. Proliferation assay of H1339 ctrl and HSF-1 k.d. cells treated with NVP-AUY922 (0, 20, 50, 75, 100 nM) for 24 h (**A**) and 48 h (**B**). Significance *** *p* ≤ 0.001. (**C**) Measurement of cell death by propidium iodide (PI) staining in H1339 ctrl and HSF-1 k.d. cells treated with NVP-AUY922 (100 nM) for 24 h. Significance ** *p* ≤ 0.01. Measurement of apoptosis induction by Annexin V (**D**) and active Caspase-3 (**E**) staining in untreated (0 nM) and NVP-AUY922 (100 nM) treated H1339 ctrl and HSF-1 k.d. cells after 24 h. Significance * *p* ≤ 0.05; ** *p* ≤ 0.01.

Targeting HSF-1 combined with inhibition of Hsp90 resulted in a concentration-dependent, significant reduction in proliferation of H1339 HSF-1 k.d. cells 24 h (Figure 2A) and 48 h (Figure 2B) after treatment. Cell death (Figure 2C) and apoptosis, as determined by Annexin V (Figure 2D) and active caspase 3 (Figure 2E) assays, was significantly increased in H1339 HSF-1 k.d. cells compared to H1339 control cells after treatment with NVP-AUY922 (100 nM). 

### 3.2. Low Hsp90 Inhibitor Concentrations Potentiate Radiosensitivity of HSF-1 k.d. Tumor Cells

**Figure 3:**
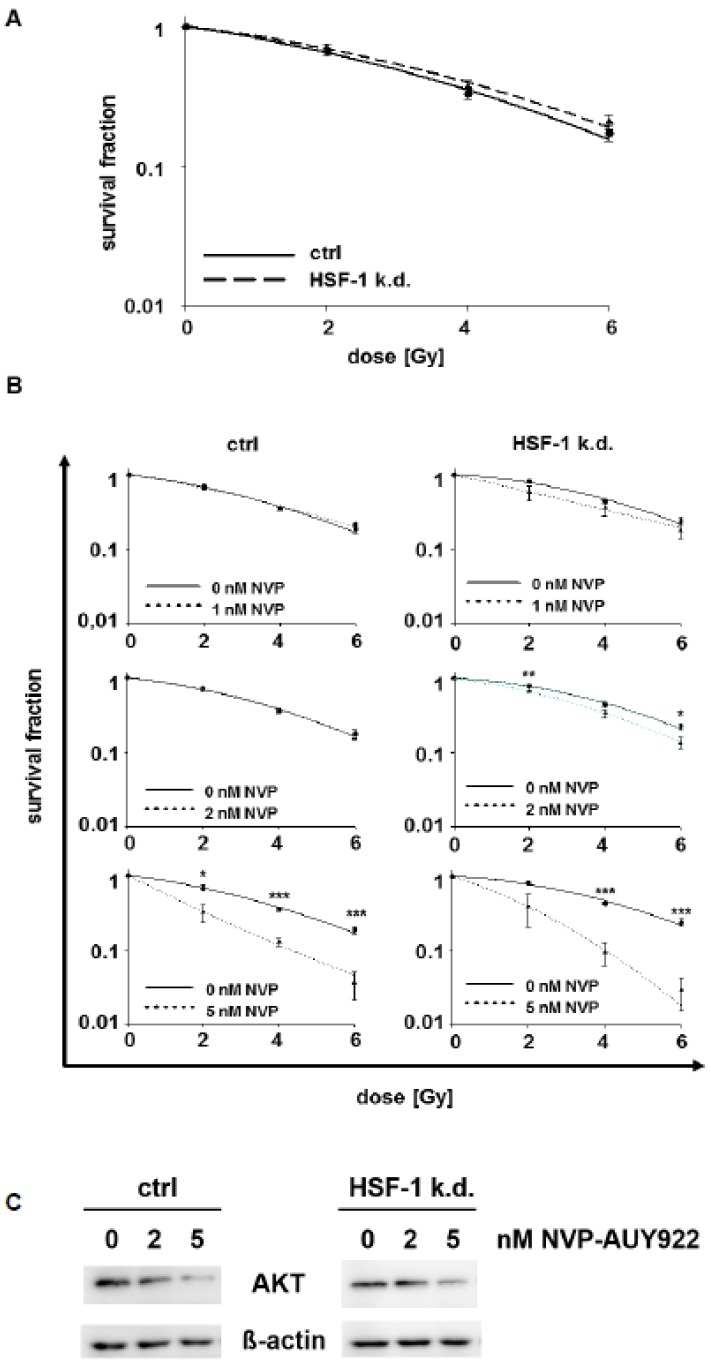
Hsp90 inhibition at low doses combined with irradiation significantly increases radiosensitivity in HSF-1 k.d. cells. (**A**) Colony forming assay of H1339 ctrl and HSF-1 k.d. cells after irradiation with 0, 2, 4, and 6Gy. (**B**) Colony forming assay of H1339 ctrl and HSF-1 k.d. cells after treatment with low concentrations of NVP-AUY922 (0, 1, 2, 5 nM) for 24 h and irradiation with 0, 2, 4, and 6Gy. Significance * *p* ≤ 0.05; ** *p* ≤ 0.01; *** *p* ≤ 0.001. (**C**) Representative immunoblot showing the expression of Akt and β-actin in H1339 cells transfected with control (ctrl) or HSF-1 shRNA (HSF-1 k.d.). Cells were treated with low concentrations of NVP-AUY922 (0, 2, 5 nM) for 24 h.

HSF-1 k.d. alone does not radiosensitize H1339 cells, as determined by clonogenic cell survival and D_50_ values (Figure 3A, Supplementary Table S1A) [34]. Therefore, we studied the combined effects of an HSF-1 k.d. and low concentrations of the Hsp90 inhibitor NVP-AUY922 (1, 2, and 5 nM). No radiosensitization was achieved in control cells by low NVP-AUY922 concentrations (up to 2 nM), whereas HSF-1 k.d. cells could be significantly radiosensitized by 2 nM NVP-AUY922 (Figure 3B, Supplementary Table S1B). A concentration of 5 nM NVP-AUY922 increased the radiosensitivity in both cell types, but the radiosensitizing effect was significantly more pronounced in HSF-1 k.d. cells. The activity of NVP-AUY922 at low concentrations (0, 2, 5 nM) was demonstrated by a downregulated expression of Akt, a client protein of Hsp90. 

### 3.3. Hsp90 Inhibition and Irradiation Increase DNA Damage Response in HSF-1 k.d. Tumor Cells

**Figure 4:**
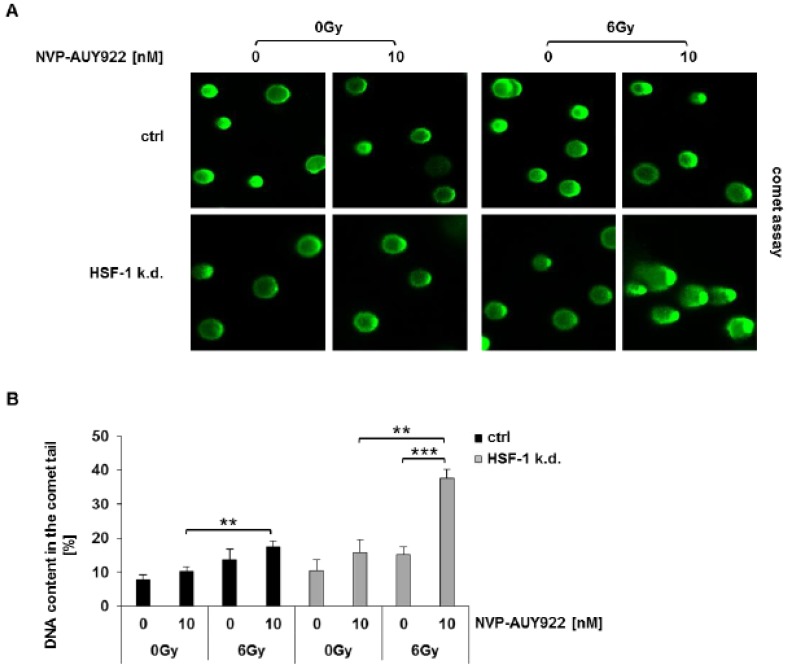
Hsp90 inhibition combined with irradiation significantly increases DNA damage in HSF-1 k.d. cells. (**A**) Comet assay 24 h after irradiation. Representative images of H1339 ctrl and HSF-1 k.d. cells after treatment with NVP-AUY922 (10 nM) for 24 h and irradiation with 0Gy and 6Gy. (**B**) Quantification of the results of the comet assay shown in (**A**). Significance ** *p* ≤ 0.01; *** *p* ≤ 0.001.

To identify the mechanisms underlying the radiosensitizing effect of a combined Hsp90 and HSF-1 inhibition in lung cancer cells, cell cycle phase distribution, apoptosis, and DNA damage response were analyzed. Neither the radiation-induced G2/M arrest (Figure S1) nor radiation-induced apoptosis (Supplementary Figure S2) were significantly impaired by NVP-AUY922 in H1339 control and HSF-1 k.d. cells. However, the DNA damage response, as determined by an alkaline comet assay, clearly demonstrated a significantly increased DNA content in the comet tail 24 h after the combined treatment with NVP-AUY922 (10 nM) and irradiation (6Gy) in HSF-1 k.d. cells (Figure 4A,B).

### 3.4. Hsp90 Inhibition Delays the Radiation-Induced DNA Double Strand Break (DSB) Repair in HSF-1 k.d. Cells

**Figure 5:**
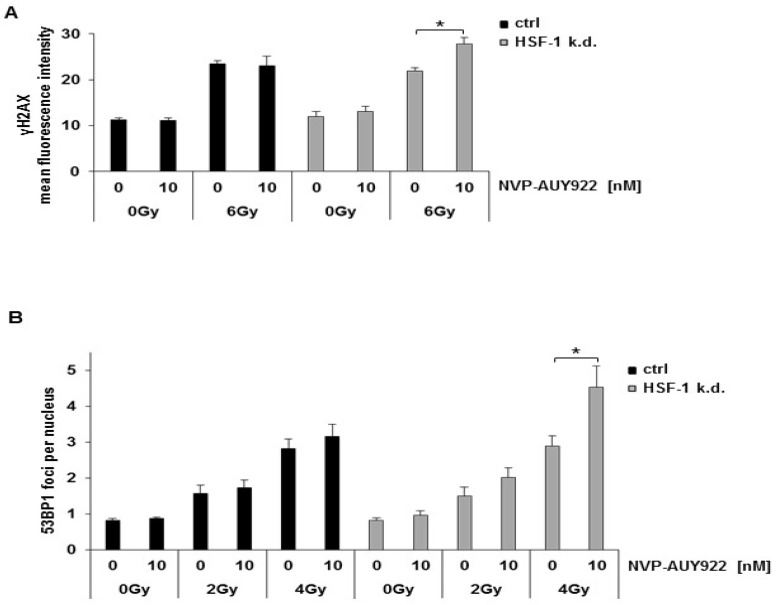
Hsp90 inhibition combined with irradiation significantly increases DNA double strand breaks in HSF-1 k.d. cells. (**A**) Mean fluorescence intensity of γH2AX measured by flow cytometry 24 h after irradiation. H1339 ctrl and HSF-1 k.d. cells after treatment with NVP-AUY922 (10 nM) for 24 h and irradiation with 0Gy and 6Gy. Significance * *p* ≤ 0.05. (**B**) Quantification of 53BP1 foci in H1339 ctrl and HSF-1 k.d. cells 24 h after irradiation. Cells were treated with NVP-AUY922 (10 nM) for 24 h following irradiation with 0Gy, 2Gy, and 4Gy. Significance * *p* ≤ 0.05.

DNA double strand breaks (DSBs) were determined in sham treated (0Gy) and irradiated (6Gy) H1339 ctrl and HSF-1 k.d. cells by measuring the expression of the DSB repair enzyme γH2AX. In line with the results of the comet assay, a combined treatment of irradiation (6Gy) and NVP-AUY922 (10 nM) led to a significant increase in the expression density of γH2AX in HSF-1 k.d. cells but not in control cells (Figure 5A). 

These results were confirmed by a 53BP1 foci assay. Since uncountable 53BP1 foci were produced by 6Gy (data not shown), the cells were irradiated only with 2 and 4Gy which caused a mean number of 1.8 and 3 repair foci per nucleus, in H1339 control and HSF-1 k.d. cells, respectively (Figure 5B). In HSF-1 k.d. cells the number of repair foci increased significantly up to 4.8 after a combined treatment with NVP-AUY922 (10 nM) and irradiation (4Gy), compared to irradiation alone (Figure 5B). These data suggest that a combined treatment significantly augments the number of DSBs in HSF-1 k.d. cells after irradiation. Since this effect was not observed 30 min after irradiation (data not shown), we hypothesize that the late DNA DSB repair might be impaired in HSF-1 k.d. cells. 

### 3.5. Hsp90 Inhibition Impairs Rad51-Mediated Homologous Recombination in Irradiated HSF-1 k.d. Cells

In eukaryotic cells, a major pathway involved in DSB repair is the homologous recombination (HR) [35,36,37]. To investigate whether NVP-AUY922 affects HR in HSF-1 k.d. cells, the formation of Rad51 foci was determined after the combined treatment regimen. 

**Figure 6:**
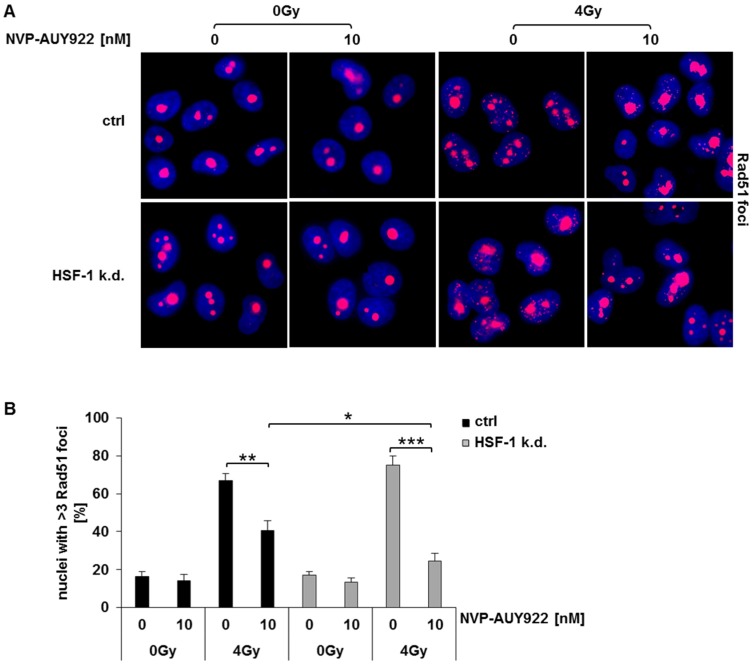
Combined treatment of Hsp90 inhibition and irradiation significantly impairs homologous recombination in HSF-1 k.d. cells. (**A**) Representative images of Rad51 foci in H1339 ctrl and HSF-1 k.d. cells 4 h after irradiation. H1339 ctrl and HSF-1 k.d. cells after treatment with NVP-AUY922 (10 nM) for 24 h and irradiation with 0Gy and 4Gy. (**B**) Quantification of Rad51 foci 4 h after irradiation. H1339 ctrl and HSF-1 k.d. cells were treated with NVP-AUY922 (10 nM) for 24 h following irradiation with 0Gy and 4Gy. Significance * *p* ≤ 0.05; ** *p* ≤ 0.01; *** *p* ≤ 0.001.

Similar to U-2 OS (osteosarcoma), A-431 (epidermoid carcinoma), and U-251 MG (glioblastoma) cells [38] Rad51 mainly resides in the nucleoli also in untreated H1339 control cells. As expected, irradiation induces an increase in Rad51 foci in control and HSF-1 k.d. cells that was slightly more pronounced in the HSF-1 k.d. cells. Representative images of a single microscopic layer are illustrated in Figure 6A. Hsp90 inhibition with NVP-AUY922 (10 nM) resulted in a significantly stronger reduction in the number of Rad51 foci in HSF-1 k.d. cells compared to control cells (Figure 6B). 

## 4. Discussion

HSF-1 is a ubiquitously expressed transcription factor that plays a central role in tumor biology (e.g., malignant transformation, carcinogenesis, and metastasis) [39,40,41,42]. HSF-1 activates the heat shock response in normal and tumor cells following stress, and thereby induces the synthesis of Hsp70 and Hsp27 [19,20,21]. Compared to normal cells, most human tumor cells exhibit an upregulated expression of HSF-1 and HSPs already under physiological conditions due to an overexpression of oncogenic proteins that require HSPs for its proper folding. Therefore, elevated HSP levels contribute to a malignant tumor phenotype and mediate resistance to chemo- and radiotherapy [6,8]. 

The inhibition of Hsp90 is considered as a promising approach to improve radiosensitivity since Hsp90 inhibition can impair a multiplicity of different oncogenic pathways in a variety of human tumor entities [43,44,45,46,47,48,49,50]. Many studies have shown that inhibition of Hsp90 induces proteasomal degradation of many important oncogenic client proteins [11,12,13,51,52].

The firstly described Hsp90 inhibitor geldanamycin and its chemical derivates 17-AAG and 17-DMAG have demonstrated radiosensitizing effects, in vitro [43,44,46,48,53,54,55]; however, poor water-solubility and severe hepatotoxicity limited their clinical applications [51,56,57,58]. The small synthetic Hsp90 inhibitor NVP-AUY922 is less toxic, shows an improved biodistribution and promising anti-tumor activity, in vitro [51,59].

Despite these advantages, similar to other clinically applied Hsp90 inhibitors, also NVP-AUY922 causes the release of HSF-1 from the Hsp90 complex and as a result HSF-1 gets activated and induces the transcription of Hsp70 and Hsp27 [23,24]. Hsp70 and Hsp27 exert cytoprotective activities and thereby can counteract the anti-tumor effects of the Hsp90 inhibition.

Previously, it was reported that NZ28—an inhibitor of several transcription factors including HSF-1, SP1, and NF-kB—can increase the radiosensitivity in human lung cancer cells. In combination with the Hsp90 inhibitor NVP-AUY922, the concentration of NZ28 could be significantly reduced to achieve radiosensitization [31,60]. With respect to these findings, we investigated the radiosensitizing effects of an HSF-1 k.d. in combination with low concentrations of NVP-AUY922 in lung cancer cells. 

In line with our previous findings, the k.d. of HSF-1 alone does not affect the radiosensitivity of H1339 cells [34]. However, in combination with the Hsp90 inhibitor NVP-AUY922, the radiosensitivity of H1339 tumor cells was significantly increased due to a down-regulation of basal and NVP-AUY922-induced levels of Hsp70 and Hsp27. These data are consistent with published data [61] demonstrating that HSF-1 depletion by shRNA can sensitize tumor cells towards high concentrations of Hsp90 inhibitors.

Our study showed for the first time, that the amount of the Hsp90 inhibitor NVP-AUY922 could be drastically reduced to the low nanomolar range when applied in combination with an HSF-1 k.d. to induce a significant radiosensitization. Since the application of Hsp90 inhibitors in the clinics is often hampered by their hepatotoxicity this finding is key for clinical applications. Our results are in line with an excellent study provided by Kudryavtsev et al. who demonstrated that a combination of Hsp90 inhibition with inhibitors of the Hsp70 response can increase radiation-induced cell death and thereby decrease post-radiation viability and clonogenicity in highly radioresistant, proliferating tumor cells. Furthermore, this group could show similar effects in proliferating tumor endothelial cells, which in turn can cause a suppression of tumor angiogenesis. In accordance to our data derived by an HSF-1 k.d., similar mechanisms of radiosensitization—including apoptosis induction and incomplete repair of DNA double strand breaks—have been demonstrated for inhibitors of the Hsp70 induction such as quercetin, triptolide, KNK437, and NZ28 [62]. 

Stingl et al. [16] indicated that NVP-AUY922 affects apoptosis as well as cell cycle-phase distribution and thereby induces radiosensitization. In line with these findings, Niewidok et al. [15] and Gandhi et al. [63] have shown that the depletion of pro-apoptotic proteins resulted in NVP-AUY922-mediated radiosensitization in lung carcinoma cells in vitro.

In our study, low concentrations of NVP-AUY922 combined with radiation did neither impact cell cycle distribution nor apoptosis in control and HSF-1 k.d. tumor cell lines (Supplementary data). Nevertheless, low Hsp90 inhibitor concentrations potentiate radiosensitization that is most likely due to an increase in DNA double-strand breaks of HSF-1 k.d. cells. In line with others showing an impairment of HR after irradiation and treatment with Hsp90 inhibitors [18,64], we also could demonstrate a significant reduction in Rad51 foci in irradiated HSF-1 k.d. cells after incubation with NVP-AUY922.

With respect to future clinical applications of a combined HSF-1 and Hsp90 inhibition to overcome radioresistance of tumor cells, presently different HSF-1 inhibiting substances such as quercetin [65], KNK437 [66], triptolide [67], NZ28 [29,31], N-amino-ethylamino derivative of colchicine (AEAC) [68], and cardenolide [69] are under investigations. However, effective concentrations in the micromolar range of quercetin, KNK437 and NZ28 are expected to exert adverse effects in patients. In contrast, triptolide and AEAC have been found to be effective in the low nanomolar range. Toxicity studies are on the way to evaluate normal tissue toxicities of these anti-Hsp70 drugs.

## 5. Conclusions

In summary, our results indicate that a combined treatment consisting of HSF-1 k.d. and low concentrations of the Hsp90 inhibitor NVP-AUY922 can significantly increase radiosensitivity in lung cancer cells. In the future, a dual targeting of HSF-1 and Hsp90 might provide a promising approach to increase the effectiveness of radiotherapy with less side effects by reducing the concentration of hepatotoxic Hsp90 inhibitors in the treatment of cancer patients.

## Supplementary Materials

The following are available online at https://www.mdpi.com/2073-4409/8/10/1166/s1, Figure S1: HSF-1 knockdown cells show no difference in cell cycle distribution after combined Hsp90 inhibition and irradiation, Figure S2: HSF-1 knockdown and control cells show no difference in apoptosis induction after combined Hsp90 inhibition and irradiation, Table S1: Summary of radiobiological parameters.
