# Supplementary material for: Radiosensitization of HSF-1 Knockdown Lung Cancer Cells by Low Concentrations of Hsp90 Inhibitor NVP-AUY922
Source: Cells. 2019 Sep 28;8(10):1166. doi: 10.3390/cells8101166 (PMC6829369; doi:10.3390/cells8101166)
Supplement: Supplementary file 1 [file cells-08-01166-s001.pdf]

Supplementary Material

Suppl. Fig. 1

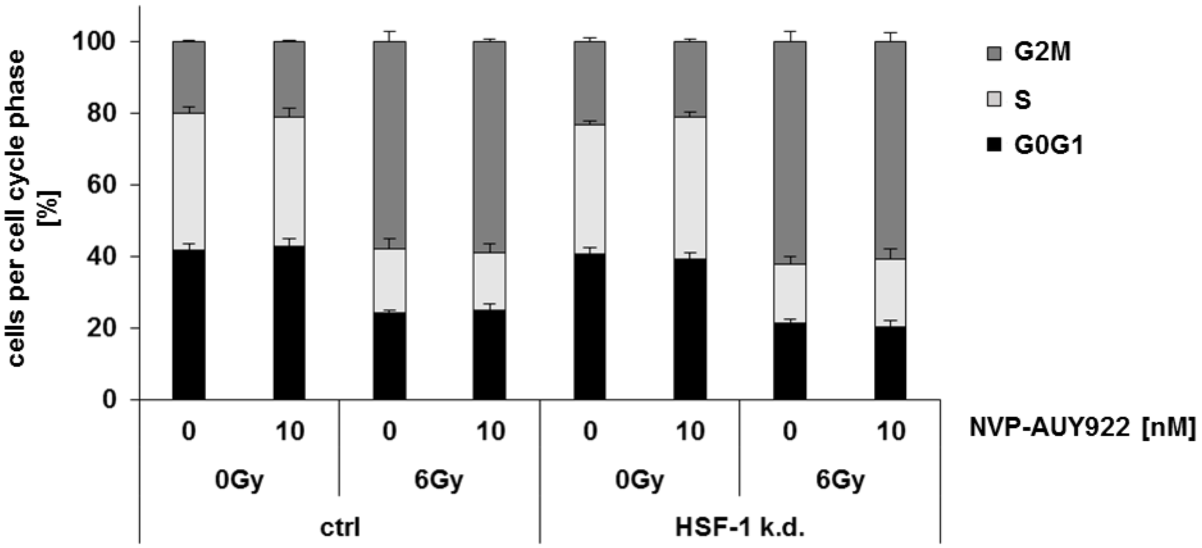

Suppl. Fig. 1 HSF-1 knockdown cells show no difference in cell cycle distribution after combined Hsp90 inhibition and irradiation

H1339 ctrl and HSF-1 k.d. cells were treated with 10nM NVP-AUY922 for 24h and irradiated with 6Gy. Cells were stained with PI to analyse cell cycle distribution 24h after irradiation.

Supplementary Material

Suppl. Fig. 2

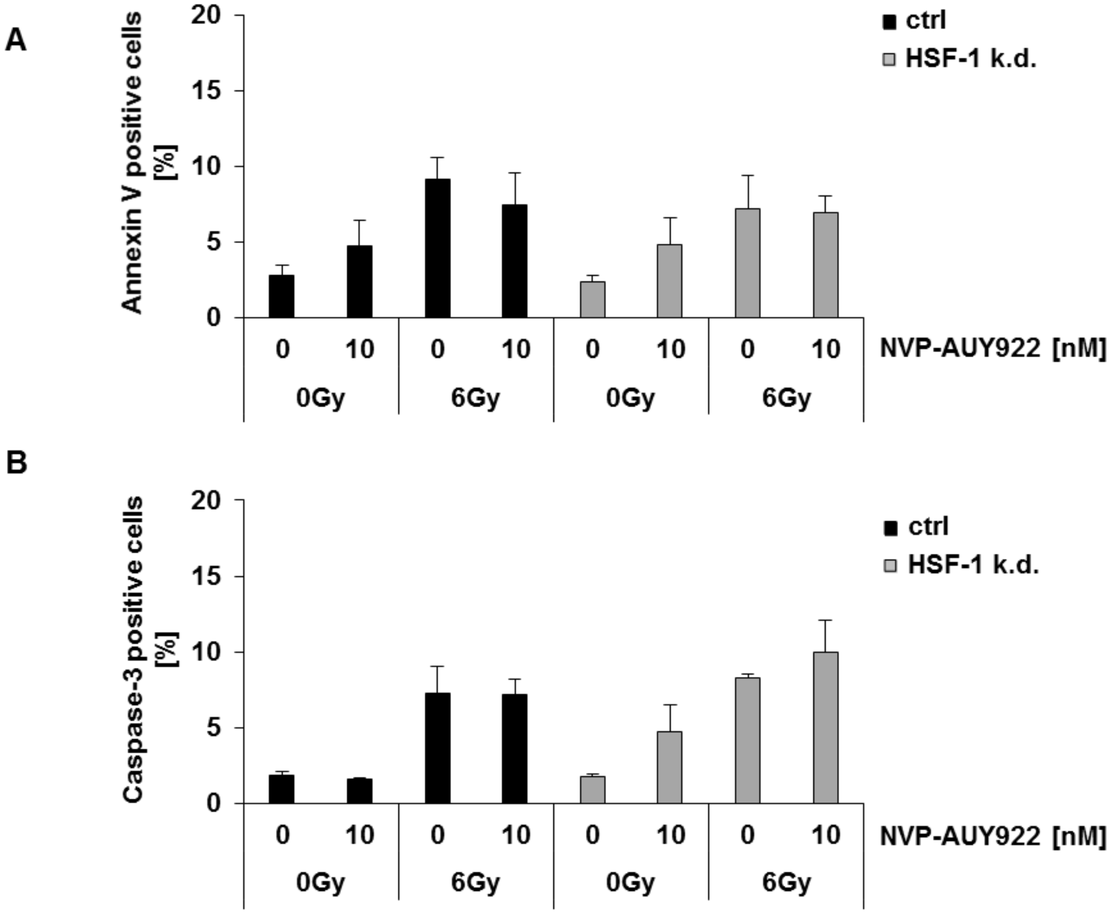

Suppl. Fig. 2 HSF-1 knockdown and control cells show no difference in apoptosis induction after combined Hsp90 inhibition and irradiation

A. Measurement of apoptosis induction 24h after irradiation by Annexin V and **B.** Caspase-3 staining of H1339 ctrl and HSF-1 k.d. cells treated with 10nM NVP-AUY922 for 24h following irradiation with 6Gy.

Supplementary Material

Supplementary Table 1: Summary of radiobiological parameters

A. Loss of HSF-1 per se has no influence on radiosensitivity. D_{50}, dose to reduce survival fraction to 50%. Sensitizing enhancement ratio (SER)=D_{50}(irradiation ctrl)/D_{50}(irradiation HSF-1 k.d.).

B. Radiosensitizing effect of Hsp90 inhibition is much more pronounced in H1339 HSF-1 k.d. cells compared to control cells. SER (D_{50}(irradiation)/D_{50}(irradiation + drug)) greater than 1.20 is indicative for radiosensitization (indicated in bold).

Tab.1

A

D_{50} dosis [Gy]		SER (50%)	
ctrl	HSF-1 k.d.	ctrl	HSF-1 k.d.
2.01	2.21	1	0.91

B

	SER (50%)	
NVP-AUY922 [nM]	ctrl	HSF-1 k.d.
0	1	1
1	1.09	**1.92**
2	0.94	**1.53**
5	**1.89**	**2.38**
